# Optimization of biotransformation processes of *Camarosporium laburnicola* to improve production yields of potent telomerase activators

**DOI:** 10.1186/s12934-024-02468-0

**Published:** 2024-07-10

**Authors:** Melis Küçüksolak, Hasan Buğra Çoban, Erdal Bedir

**Affiliations:** 1 Department of Bioengineering, Faculty of Engineering, İzmir Institute of Technology, Urla, İzmir, 35433 Turkey; 2 İzmir International Biomedicine and Genome Institute, Dokuz Eylül University, Balçova, İzmir, 35340 Turkey

**Keywords:** Whole-cell biotransformation, Endophytic fungi, Process optimization, Anti-aging, Telomerase activator

## Abstract

**Background:**

Telomerase activators are promising agents for the healthy aging process and the treatment/prevention of short telomere-related and age-related diseases. The discovery of new telomerase activators and later optimizing their activities through chemical and biological transformations are crucial for the pharmaceutical sector. In our previous studies, several potent telomerase activators were discovered via fungal biotransformation, which in turn necessitated optimization of their production. It is practical to improve the production processes by implementing the design of experiment (DoE) strategy, leading to increased yield and productivity. In this study, we focused on optimizing biotransformation conditions utilizing *Camarosporium laburnicola*, a recently discovered filamentous fungus, to afford the target telomerase activators (E-CG-01, E-AG-01, and E-AG-02).

**Results:**

DoE approaches were used to optimize the microbial biotransformation processes of *C. laburnicola*. Nine parameters were screened by Plackett-Burman Design, and three significant parameters (biotransformation time, temperature, shaking speed) were optimized using Central Composite Design. After conducting validation experiments, we were able to further enhance the production yield of target metabolites through scale-up studies in shake flasks (55.3-fold for E-AG-01, 13-fold for E-AG-02, and 1.96-fold for E-CG-01).

**Conclusion:**

Following a process optimization study using *C. laburnicola*, a significant increase was achieved in the production yields. Thus, the present study demonstrates a promising methodology to increase the production yield of potent telomerase activators. Furthermore, *C*. *laburnicola* is identified as a potential biocatalyst for further industrial utilization.

**Supplementary Information:**

The online version contains supplementary material available at 10.1186/s12934-024-02468-0.

## Background

Telomerase activation is a promising strategy for treating diseases that result from telomere loss. Telomere shortening is a natural part of aging, and this situation can increase the risk of several age-related disorders, namely Alzheimer’s disease, macular degeneration, myelodysplastic syndrome, Parkinson’s disease, and type 2 diabetes [1, 2]. However, telomere-related diseases are not limited to age-related conditions. Congenital dyskeratosis, aplastic anemia, and pulmonary fibrosis are among the diseases that can also be caused by telomere dysfunction [3, 4]. Therefore, telomerase enzyme activators are shown to be promising agents for developing new therapies that can help mitigate the effects of telomere loss and related conditions [5–8].

Cycloastragenol is a very significant telomerase activator, and it has been in the market as an anti-aging dietary supplement with the trade name TA-65. Cycloastragenol, possessing a cycloartane-type framework, is only biosynthesized in *Astragalus* species. The use of cycloastragenol as a telomerase activator in regenerative medicine and degenerative diseases has been demonstrated by many studies [9–15]. Additionally, the entry of cycloastragenol and derivatives into clinical trials for Alzheimer’s disease and metabolic syndrome (NCT numbers: 02531334 and 02530255, respectively) indicates the potential of telomerase activators towards degenerative diseases. In 2022, two new clinical studies were initiated using *Astragalus membranaceus* in cognitive impairment and parkinsonism (NCT numbers: 05578443 and 05506891, respectively).

Biotransformation is the biochemical reaction of living systems that alters molecules. This methodology utilizing highly diverse enzyme systems is used to improve the bioactivity profiles of precursor molecules and develop molecular libraries for structure-activity relationship studies. It is also applied to predict mammalian drug metabolism. In addition, biotransformation is a highly effective tool in producing compounds that are challenging to synthesize using conventional chemical processes [16–19].

Biotransformation is divided into two classes according to the biocatalyst used. Microorganisms, plants, or mammalian cells perform biocatalysis in whole-cell systems, while isolated enzyme systems are used in the enzymatic biotransformation process [20, 21]. Whole-cell biotransformation has many advantages over enzymatic transformation. Whole-cell systems provide a stable environment for enzymes, and thus, they can be used for extended periods. They are also advantageous in regenerating cofactors such as NAD(P)H and ATP. These systems contain multiple enzymatic pathways; they provide optimum enzyme concentrations and energy levels, minimize activity loss, and allow multi-step transformations. They also offer a less costly process by eliminating the need for isolation and purification of enzymes. Because of these advantages, whole-cell systems are preferred more than enzymatic systems in industrial processes [20, 22–24].

Nevertheless, whole-cell biotransformation is often affected directly and indirectly by many environmental factors, including the composition of media components and process parameters, such as pH and temperature. Therefore, optimizing whole-cell biotransformation processes is of great importance in transferring studies from laboratory to industrial scale [25, 26]. Two approaches are generally used in optimizing these processes: one factor at a time (OFAT) and the design of experiment (DoE). In the OFAT approach, the limits of one variable are changed while the other variables are kept constant. This method requires a lot of workload, takes time, and allows one-dimensional analysis, and it cannot generally guarantee that the optimum point has been determined [27, 28]. As testing all possibilities is impractical, the OFAT approach may not be sufficient for microbial processes with many parameters. In addition to needing a lot of experimentation, optimum points can only be determined if the limits are chosen correctly. On the other hand, statistical experimental design approaches, known as DoE, allow the optimum points to be determined accurately since second and higher-order models can be developed [26, 29].

Plackett-Burman Design (PBD) is a method used to eliminate parameters that are not effective in production reliably and practically without the need for many experiments [30]. PBD is very useful when the effects of many variables are studied, but only some of these variables are effective. This method allows us to determine the effects of *n* variables on the process by performing *n* + 1 trials with a two-level fractional factorial design. Although this method ignores the interaction between parameters, it allows the reliable selection of a small number of effective parameters from many variables. PBD generally results in three or four significant factors. After that, significant factors are brought into the response surface methodology (RSM) for further optimization [26, 27].

RSM is a combination of mathematical and statistical techniques used to develop new processes or optimize existing processes to reveal the interaction between parameters and the dual or individual effects of parameters on the process with a second or higher-order equation [31]. The application of RSM generally reduces the cost of expensive analysis methods and increases production yields. RSM is an effective tool for optimizing medium compositions, conditions of enzymatic hydrolysis, and fermentation processes [32].

Central Composite Design (CCD) is the most widespread method for developing a quadratic model among RSM approaches. CCD consists of a factorial point (± 1), an axial point in which experimental points are at a distance from its center (±α), and a central point (0). The axial point (α) and the number of trials at the center point increase the number of levels and give the experimental design flexibility. In addition, CCD allows one to know what effect the factors had on the response if one goes beyond or below the chosen levels of factors. The axial point and the number of trials at the center point influence the estimation accuracy [33, 34].

DoE approaches have been used in microbial biotransformation studies, and a combination of PBD and RSM has been successfully applied to increase production yields by optimizing parameters [26, 27, 35, 36].

**Fig. 1:**
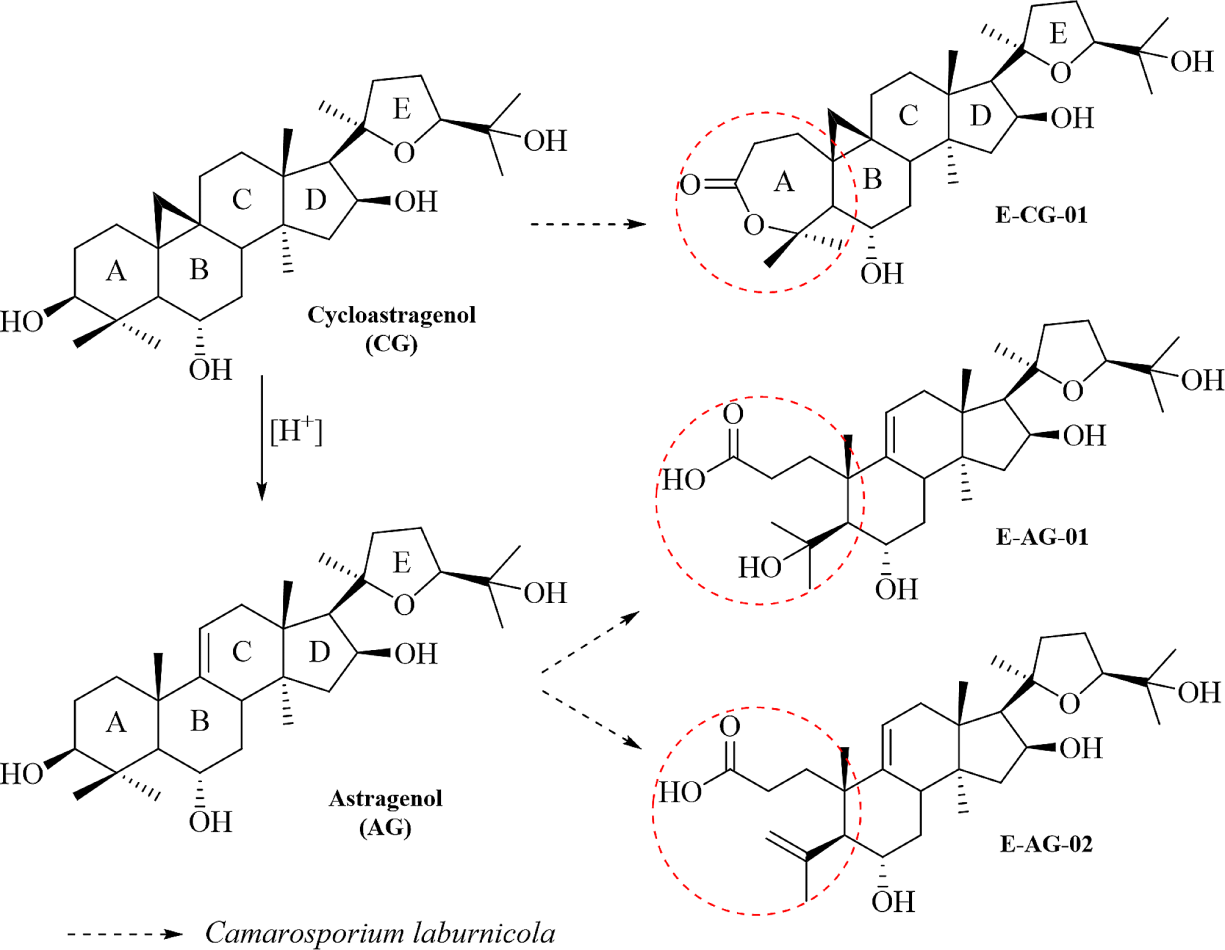
Chemical structures of starting compounds (CG and AG) and target metabolites (E-CG-01, E-AG-01 and E-AG-02)

In our previous studies, we carried out biotransformation studies on cycloartanes utilizing *Astragalus* endophytic fungi to discover new telomerase activators [37–39]. In the telomerase activation screening panel, sixteen compounds showed activity ranging from 1.2 to 11.3-fold at doses of 0.5 to 300 nM compared to the control cells treated with DMSO. The major modifications leading to increased bioactivity occurred in ring A (Fig. 1, Table S1), catalyzed by *Camarosporium laburnicola*, an endophytic fungus isolated from *Astragalus angustifolius* and employed for the first time for biotransformation studies [37]. Since no effort was made to increase metabolite production or to search for alternative methods for high efficiency and productivity of newly discovered telomerase activators, in the current study, we made a further attempt to optimize the biotransformation conditions to increase the production efficiency of the potent telomerase activators (viz. E-CG-01, E-AG-01, and E-AG-02) to be utilized in preclinical and clinical studies.

## Results

### Growth curve of *Camarosporium laburnicola*

**Fig. 2:**
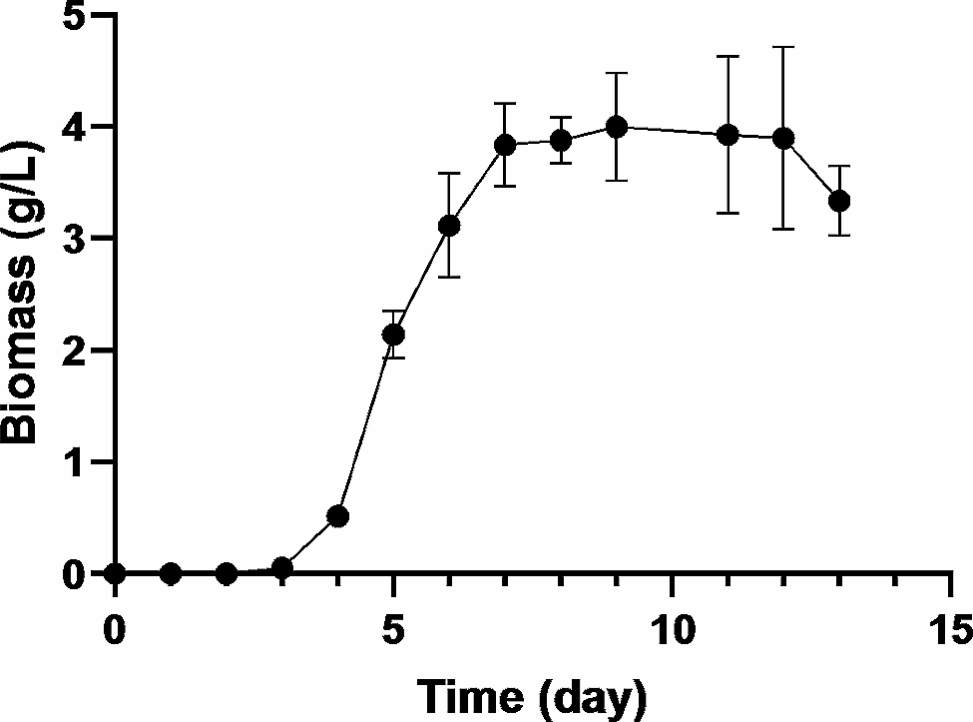
Growth curve of *Camarosporium laburnicola*

A growth kinetics experiment was performed for *C. laburnicola* since no kinetic data was available for the fungus. The growth curve is presented in Fig. 2. In submerged culture conditions, the lag phase of *C. laburnicola* was completed on the 3^rd^ day, the log phase was completed on the 8^th^ day, and the fungal metabolism passed to the stationary phase. Based on the growth curve, the µ_max_ and the doubling time were calculated as 0.059 h^-1^ and 11.67 h, respectively.

### Plackett-Burman Design

Table 1 shows the results of a 12-run PBD conducted to determine which process parameters contributed to higher production yields of potent telomerase activators among nine parameters. The responses were converted into target metabolite per liter to compare the results. The most effective parameters for producing E-AG-01 and E-AG-02 were biotransformation time, temperature, and shaking speed. The total contribution of these parameters was calculated as 73.89% for E-AG-01 and 76.76% for E-AG-02; the other parameters had minor effects on these metabolites (Table 2). For E-CG-01, biotransformation time and temperature were the two parameters with the highest effect in the biotransformation process. The total effect of these two parameters was 48.76% (Table 2). Another important parameter is the substrate feeding time, with a contribution rate of 11.54%. However, as we observed an increase in metabolite diversity during preliminary experiments by changing the substrate feeding time, we kept this parameter constant for further experiments (Fig. S1). Furthermore, shaking speed was selected for optimization experiments of E-CG-01, a mutual parameter with E-AG-01 and E-AG-02.

**Table 1:** Plackett–Burman Design matrix and results

Run	SFT (day)	BT (day)	T (°C)	pH	WV (mL)	SS (rpm)	IS (v/v %)	SC (w/v %)	TC (v/v %)	D1	D2	E-AG-01^a^ (mg/L)	E-AG-02^a^ (mg/L)	E-CG-01^b^ (mg/L)
1	4	9	20	6.6	70	210	2	0.01	0	1	-1	14.80	41.50	32.33
2	2	9	30	4.6	70	210	20	0.01	0	-1	1	10.31	19.48	33.15
3	4	3	30	6.6	30	210	20	0.03	0	-1	-1	0.14	1.96	3.96
4	2	9	20	6.6	70	150	20	0.03	0.2	-1	-1	16.58	41.96	55.56
5	2	3	30	4.6	70	210	100	0.03	0.2	1	-1	0	0	0
6	2	3	20	6.6	30	210	20	0.01	0.2	1	1	6.38	8.60	25.25
7	4	3	20	4.6	70	150	20	0.03	0	1	1	1.39	10.13	34.08
8	4	9	20	4.6	30	210	2	0.03	0.2	-1	1	22.05	54.53	21.12
9	4	9	30	4.6	30	150	20	0.01	0.2	1	-1	0.28	2.62	10.44
10	2	9	30	6.6	30	150	2	0.03	0	1	1	0.10	1.68	36.57
11	4	3	30	6.6	70	150	2	0.01	0.2	-1	1	0	0	1.02
12	2	3	20	4.6	30	150	2	0.01	0	-1	-1	0	0.31	24.03

SFT: Substrate feeding time; BT: Biotransformation time; T: Temperature; pH: Initial pH; WV: Working volume; SS: Shaking speed; IS: Inoculum size; SC: Substrate concentration; TC: Tween 80 concentration; D1: Dummy 1; D2: Dummy 2; Responses: E-AG-01, E-AG-02, and E-CG-01

^a^Astragenol was fed into a nutrient medium

^b^Cycloastragenol was fed into a nutrient medium

**Table 2:** Plackett–Burman Design % contribution of the parameters

	SFT (day)	BT (day)	T (°C)	pH	WV (mL)	SS (rpm)	IS (v/v %)	SC (w/v %)	TC (v/v %)	D1	D2
%Cont. for E-AG-01^a^	0.30	33.75	26.72	0.17	2.09	13.42	0.03	0.83	3.63	7.00	0.74
%Cont. for E-AG-02^a^	2.68	36.31	31.59	0.13	3.37	8.86	0.31	2.65	1.90	5.19	0.06
%Cont. for E-CG-01^b^	11.54	22.88	25.88	2.28	2.72	4.74	5.05	1.42	5.79	0.00007	1.39

SFT: Substrate feeding time; BT: Biotransformation time; T: Temperature; pH: Initial pH; WV: Working volume; SS: Shaking speed; IS: Inoculum size; SC: Substrate concentration; TC: Tween 80 concentration; D1: Dummy 1; D2: Dummy 2; %Cont.: %Contribution. Responses: E-AG-01, E-AG-02, and E-CG-01

^a^Astragenol was fed into a nutrient medium

^b^Cycloastragenol was fed into a nutrient medium

### Central composite design

The parameters and results of CCD experiments are given in Table 3. According to CCD experiments, productions of telomerase activators were fit to third-order polynomial equations (Eqs. 1, 2, and 3).

1 $$\begin{array}{l}E - AG - 01{\rm{ }}\left( {mg/L} \right) = - 286.9616 - 34.0226A + 11.4478B + \\2.3415C + 1.7506AB + 3.2807{A^2} - 0.3410{B^2} - 0.0064{C^2} - 0.1595{A^2}B\end{array}$$

2 $$\begin{array}{l}E - AG - 02{\rm{ }}\left( {mg/L} \right) = - 612.6694 - 25.7779A + 26.6191B\\+ 3.7440C + 1.8549AB + 3.3630{A^2} - 0.6465{B^2} - 0.0104{C^2} - 0.1936{A^2}B\end{array}$$

3 $$\begin{array}{l}E - CG - 01{\rm{ }}\left( {mg/L} \right) = - 9.5178 - 1.7101A - 11.4628B + 1.8161C{\rm{ }}\\+ {\rm{ }}4.0259AB - 0.4323AC - 0.0437BC - 0.5648{A^2} + 0.26378{B^2} + \\0.0068ABC - 0.1315{A^2}B{\rm{ }} + 0.0198{A^2}C - 0.0737A{B^2}\end{array}$$

where A, B, and C are biotransformation time (day), temperature (°C), and shaking speed (rpm), respectively.

**Table 3:** CCD matrix and with experimental responses

Run	BT (day)	T (°C)	SS (rpm)	E-AG-01^a^ (mg/L)	E-AG-02^a^ (mg/L)	E-CG-01^b^ (mg/L)
1	6	17	180	14.12	26.02	30.56
2	9	30	150	0	0	0
3	3	20	210	18.85	18.84	25.30
4	3	30	210	0	0	0
5	9	20	210	36.56	44.39	24.90
6	6	25	180	34.14	62.18	20.82
7	3	20	150	16.86	18.85	19.24
8	9	30	210	2.78	2.63	1.76
9	1	25	180	5.21	4.50	9.17
10	3	30	150	0	0	0
11	11	25	180	21.18	37.73	23.24
12	6	25	130	13.08	31.42	25.78
13	6	25	180	35.27	57.42	24.49
14	6	25	180	27.5	54.00	25.52
15	9	20	150	29.26	48.85	32.13
16	6	25	230	23.15	45.05	18.27
17	6	33	180	0	0	0
18	6	25	180	31.78	54.10	19.32

BT: Biotransformation time; T: Temperature; SS: Shaking speed. Responses: E-AG-01, E-AG-02, and E-CG-01

^a^Astragenol was fed into a nutrient medium

^b^Cycloastragenol was fed into a nutrient medium

The variance analysis (ANOVA) and regression analysis results for E-CG-01, E-AG-01, and E-AG-02 are denoted in Tables 4 and 5, and 6. The model’s *F* and *p* values indicate model’s success in predicting target metabolite production. The non-significant lack of fit indicates that the results sufficiently fit with the model. The *p*-values less than 0.05 are considered significant, and values greater than 0.1 are non-significant model terms.

**Fig. 3:**
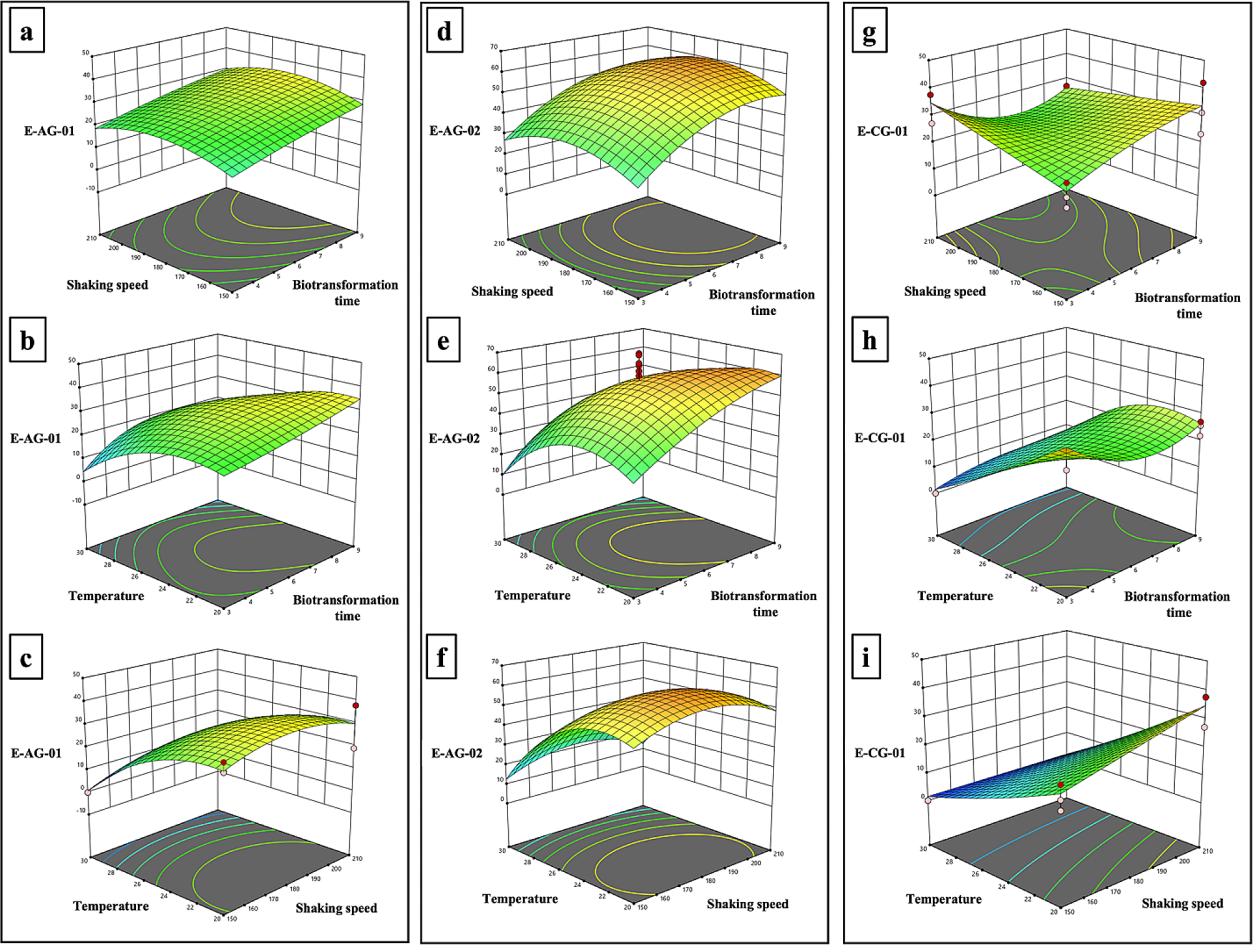
Mutual factor interactions among temperature, biotransformation time, and shaking speed on E-AG-01, E-AG-02, and E-CG-01 production. Three-dimensional response surface plot E-AG-01 production (**a**) as a function of shaking speed and biotransformation time, (**b**) as a function of temperature and biotransformation time and (**c**) as a function of temperature and shaking speed. Three-dimensional response surface plot E-AG-02 production (**d**) as a function of shaking speed and biotransformation time, (**e**) as a function of temperature and biotransformation time and (**f**) as a function of temperature and shaking speed. Three-dimensional response surface plot E-CG-01 production (**g**) as a function of shaking speed and biotransformation time, (**h**) as a function of temperature and biotransformation time, and (**i**) as a function of temperature and shaking speed

The *p*-values were < 0.0001, and *F* values (E-AG-01: 33.62, E-AG-02: 78.80, E-CG-01: 12.84) suggested that models are significant and are likely to occur by chance, with a very low probability of 0.01%. According to ANOVA analyses, A, B, AB, A^2^, B^2^, C^2^, and A^2^B are variables that have a statistically significant effect for E-AG-01 (Table 4) and E-AG-02 (Table 5), whereas A, B, C, A^2^, B^2^, ABC, A^2^B, A^2^C, AB^2^ are significant terms for E-CG-01 (Table 6). The closer R^2^ values indicate a better correlation between the predicted and experimental values, and R^2^ was calculated as 0.8622 for E-AG-01, 0.9361 for E-AG-02, and 0.8149 for E-CG-01 in our models. The signal-to-noise ratio was measured by Adeq. Precision values (E-AG-01: 15.0576, E-AG-02: 22.1028, E-CG-01: 10.8299) and greater than four indicate that these models can navigate the design space. The mutual effect of parameters on the production of potent telomerase activators can be interpreted by the three-dimensional graphs of CCD given in Fig. 3.

**Table 4:** ANOVA of CCD experiments for E-AG-01

Source	Sum of squares	df	Mean square	*F*-value	*p*-value	
Model	7990.61	8	998.83	33.62	< 0.0001	significant
A-Biotransformation time	576.96	1	576.96	19.42	< 0.0001	
B-Temperature	299.11	1	299.11	10.07	0.0028	
C-Shaking speed	19.87	1	19.87	0.6687	0.4180	
AB	136.81	1	136.81	4.60	0.0376	
A²	1516.41	1	1516.41	51.04	< 0.0001	
B²	2728.84	1	2728.84	91.84	< 0.0001	
C²	1148.21	1	1148.21	38.65	< 0.0001	
A²B	501.62	1	501.62	16.88	0.0002	
Residual	1277.59	43	29.71			
Lack of Fit	65.90	6	10.98	0.3354	0.9138	not significant
Pure Error	1211.69	37	32.75			
Cor Total	9268.21	51				
R²	0.8622					
Adjusted R²	0.8365					
Predicted R²	0.8101					
Adeq Precision	15.0576					

**Table 5:** ANOVA of CCD experiments for E-AG-02

Source	Sum of squares	df	Mean square	*F*-value	*p*-value	
Model	25712.46	8	3214.06	78.80	< 0.0001	significant
A-Biotransformation time	2940.10	1	2940.10	72.08	< 0.0001	
B-Temperature	1015.63	1	1015.63	24.90	< 0.0001	
C-Shaking speed	3.98	1	3.98	0.0977	0.7561	
AB	1129.18	1	1129.18	27.68	< 0.0001	
A²	6639.09	1	6639.09	162.78	< 0.0001	
B²	9808.81	1	9808.81	240.49	< 0.0001	
C²	2980.76	1	2980.76	73.08	< 0.0001	
A²B	739.42	1	739.42	18.13	0.0001	
Residual	1753.83	43	40.79			
Lack of Fit	162.92	6	27.15	0.6315	0.7041	not significant
Pure Error	1590.91	37	43.00			
Cor Total	27466.29	51				
R²	0.9361					
Adjusted R²	0.9243					
Predicted R²	0.9134					
Adeq Precision	22.1028					

**Table 6:** ANOVA of CCD experiments for E-CG-01

Source	Sum of squares	df	Mean square	*F*-value	*p*-value	
Model	6598.81	12	549.90	12.84	< 0.0001	significant
A-Biotransformation time	482.73	1	482.73	11.27	0.0019	
B-Temperature	737.68	1	737.68	17.23	0.0002	
C-Shaking speed	231.37	1	231.37	5.40	0.0260	
AB	0.4907	1	0.4907	0.0115	0.9154	
AC	105.24	1	105.24	2.46	0.1260	
BC	3.63	1	3.63	0.0847	0.7727	
A²	219.93	1	219.93	5.14	0.0297	
B²	667.49	1	667.49	15.59	0.0004	
ABC	202.42	1	202.42	4.73	0.0365	
A²B	292.38	1	292.38	6.83	0.0131	
A²C	240.18	1	240.18	5.61	0.0235	
AB²	226.06	1	226.06	5.28	0.0277	
Residual	1498.70	35	42.82			
Lack of Fit	182.29	2	91.14	2.28	0.1177	not significant
Pure Error	1316.42	33	39.89			
Cor Total	8097.51	47				
R²	0.8149					
Adjusted R²	0.7515					
Predicted R²	0.7060					
Adeq Precision	10.8299					

Three replicate verification experiments were carried out for each metabolite to validate CCD models. Tables 7 and 8, and 9 show experimental conditions and results.

**Table 7:** Validation experiments of CCD for E-AG-01

No	BT (day)	T (ºC)	SS (rpm)	R (Predicted) (mg/L)	R (Experimental) (mg/L)	95% CI low (mg/L)	95% CI high (mg/L)
1	3	20	210	17.28	10.16	5.42	29.13
2	8	22	180	34.15	35.47	22.74	45.56
3	6	22	180	31.54	31.62	20.08	43.00

BT: Biotransformation time; T: Temperature; SS: Shaking speed. R: Response, E-AG-01 concentration

**Table 8:** Validation experiments of CCD for E-AG-02

No	BT (day)	T (ºC)	SS (rpm)	R (Predicted) (mg/L)	R (Experimental) (mg/L)	95% CI low (mg/L)	95% CI high (mg/L)
1	3	20	210	19.44	13.03	5.54	33.33
2	8	22	180	60.85	59.19	47.48	74.22
3	6	22	180	55.89	57.43	42.46	69.32

BT: Biotransformation time; T: Temperature; SS: Shaking speed. R: Response, E-AG-02 concentration

**Table 9:** Validation experiments of CCD for E-CG-01

No	BT (day)	T (ºC)	SS (rpm)	R (Predicted) (mg/L)	R (Experimental) (mg/L)	95% CI low (mg/L)	95% CI high (mg/L)
1	3	20	210	34.71	35.19	18.64	50.79
2	8	22	180	29.06	40.92	15.36	42.76
3	6	22	180	25.59	24.29	11.84	39.34

BT: Biotransformation time; T: Temperature; SS: Shaking speed. R: Response, E-CG-01 concentration

### Scale-up studies

**Fig. 4:**
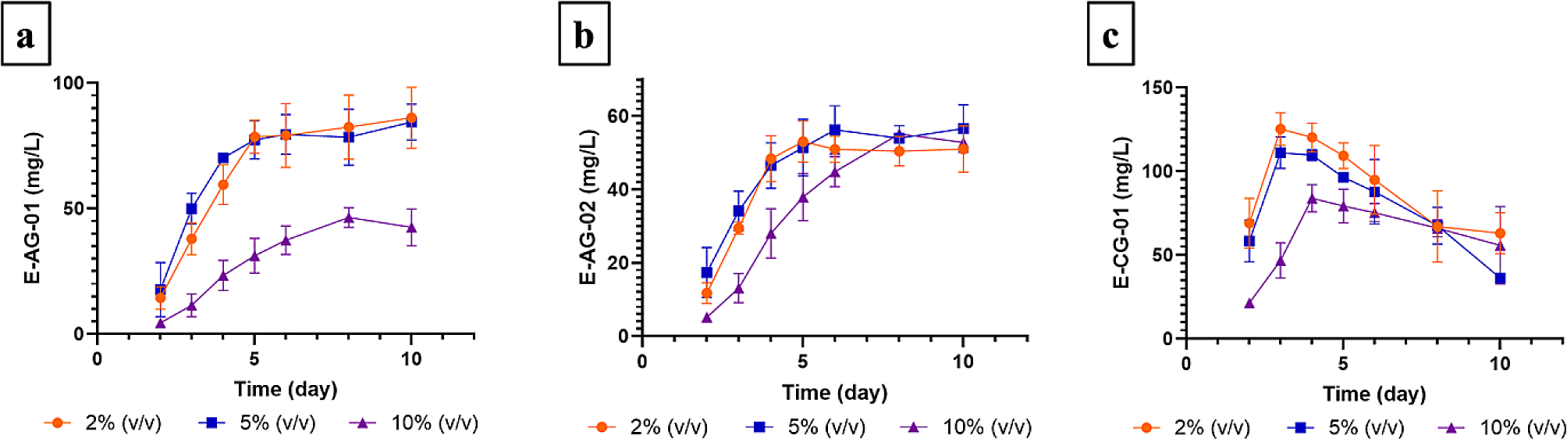
Production kinetics of (**a**) E-AG-01, (**b**) E-AG-02, and (**c**) E-CG-01 using *C. laburnicola*. Inoculation was performed with an inoculum size of 2%, 5% and 10%

The time-dependent measurement of target metabolites in 5 L flasks is given in Fig. 4. The concentrations of E-AG-01 and E-AG-02 increased rapidly from day 2 to 4 and reached the highest concentration on day 10 (2% inoculum size) and day 8 (10% inoculum size), respectively (Fig. 4a and b). The maximum concentrations for E-AG-01 and E-AG-02 were 86.21 mg/L and 55.18 mg/L, respectively. For the E-CG-01, the highest concentration (125.32 mg/L) was found in the broth on day 3, and its concentration decreased starting from day 4 for 2% inoculum size (Fig. 4c).

## Discussion

Whole-cell biotransformation systems are an important tool in the production of pharmaceuticals due to their ability to catalyze cofactor-dependent reactions and multi-step reactions with the combination of enzymes, as well as their low costs. In addition to these advantages, whole-cell biotransformation has disadvantages, such as low selectivity and undesirable side reactions. Fortunately, several strategies, such as synthetic biology and metabolic engineering toolboxes, have been developed to avoid these disadvantages. In this way, industrial use of whole-cell biotransformation is possible [23, 40–42]. In addition to these strategies, process optimization with the design of experiment approach emerges as a lower-cost strategy [26, 31, 32].

**Fig. 5:**
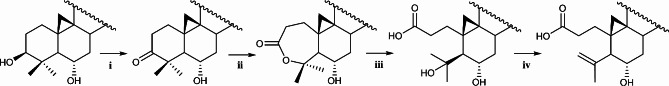
The proposed biochemical reaction chain for modifications occurring in the A ring. (**i**) Dehydrogenase enzyme, (**ii**) Baeyer Villiger Monooxygenase (BVMO) enzyme, (**iii**) Lactone hydrolase enzyme, (**iv**) Lyase enzyme

In our previous studies, a newly discovered fungus, *Camarosporium laburnicola*, has played an important role in producing potent telomerase activators by catalyzing the multi-step enzymatic reaction (Fig. 5) [37, 39]. However, we observed that these metabolites rapidly converted into each other through these biochemical reaction chains, which resulted in low yields. For this reason, performing optimization studies to produce high amounts of these metabolites becomes necessary for sustainability and the transition to industrial production of these metabolites. Additionally, since the starting compounds are costly the importance of process optimization is worthy. Therefore, we used the design of experiment approach to improve the yields of our target metabolites. *C. laburnicola*, with mostly silent secondary metabolite pathways, providing narrow metabolite diversity as a biocatalyst, and its potential to afford bioactive transformation products, especially towards telomerase activation, were the overriding basis for selecting this fungus for further studies.

PBD was used to screen the parameters that could be effective in the biotransformation process and to subtract those that were not statistically effective. The parameters screened with PBD were temperature, pH, inoculum size, substrate concentration, Tween 80 concentration, substrate feeding time, biotransformation time, working volume, and shaking speed. To determine the lower and upper limits of the substrate feeding time and biotransformation time, a growth curve was generated for *C. laburnicola* based on dry weight (Fig. 2). According to the growth curve of *C. laburnicola*, the lag phase was completed on the 3^rd^ day, followed by the log phase on the 8^th^ day. Subsequently, the fungal metabolism transitioned into the stationary phase. In our preliminary studies with *C. laburnicola*, substrate feeding was performed on the 3^rd^ day, and the biotransformation studies lasted 4–10 days, and the substrate was completely consumed in these conditions. From this point of view, we suggested that the reactions catalyzed by *C. laburnicola* occur during the growth phase. To confirm this observation, the lower limit was chosen as the 2^nd^ day, and the upper limit was selected as the 4^th^ day for the substrate feeding time. The lower limit for the biotransformation time was determined as three days, and the upper limit was nine days. According to PBD results, important factors influencing the production of E-AG-01, E-AG-02, and E-CG-01 were biotransformation time, temperature, and shaking speed. These variables were selected for statistical optimization based on a CCD design.

CCD trials were carried out to find the optimum values for the three parameters (temperature, biotransformation time, and shaking speed), which were determined to be effective according to the PBD trials. 2^3^ full factorial CCD was selected to evaluate the individual and combined effects of temperature, biotransformation time, and shaking speed. In these trials, the lower and upper limits of the parameters were selected as 17–33 °C for the temperature, 1–11 days for the biotransformation time, and 130–230 rpm for the shaking speed to evaluate the effects in a wider range.

The experimental results were interpreted through three-dimensional graphs obtained with the Design Expert 12. The following results can be summarized for target molecules from the three-dimensional graphs (Fig. 3). As seen in Fig. 3b, c, e, f and h, and 3i, temperature has a negative effect on the production efficiency of all three metabolites, and this parameter should be kept between 20 and 23 °C. Moreover, the interaction of biotransformation time with shaking speed influences the production yield of E-CG-01; high agitation speed and short biotransformation time or vice versa increase the yield (Fig. 3g). For E-AG-01 and E-AG-02, long biotransformation time increases the production yield of AG derivatives (Fig. 3a, b and d, and 3e). Additionally, the central point of shaking speed (180 rpm) provides the highest yield in producing AG derivatives (Fig. 3a, c and d, and 3f).

The optimization tool of Design Expert 12 suggested that the optimum temperature and shaking speed for AG-derived metabolites were 22 °C and 180 rpm, and biotransformation time was 9 days and 8 days for E-AG-01 and E-AG-02, respectively. Under these conditions, the highest concentration was 35.17 mg/L for E-AG-01 and 61 mg/L for E-AG-02. While the E-AG-01 yield increased by 22.56-fold, the E-AG-02 yield improved by 14.37-fold compared to non-optimized conditions. For E-CG-01, the maximum yield was reached at two conditions (20 °C, 150 rpm, 9 days, and 20 °C, 210 rpm, 3 days). Considering that time is an important parameter in industrial production, 20 °C, 210 rpm, and 3 days were determined as the optimum conditions for maximum production. However, a 0.54-fold decrease was obtained compared to non-optimized conditions for E-CG-01. According to validation experiments, all trials are within the 95% confidence interval. In addition, all the experimental data were close to the predicted values. These results show that the models can predict the production of target metabolites.

During the optimization studies, we observed that the fungus developed pellet structures. Growth of filamentous fungi can display dispersed or aggregated pellet structures in submerged culture [43, 44]. Dispersed mycelia result in high biomass concentrations, highly viscous fermentation media, and restrictions in gas-liquid transfer, while complex pellets cause limitations in the mass transfer of substrates, products, and oxygen [45]. Therefore, the morphology of fungal growth is highly linked with process control and productivity and depends on the type of production [43, 44, 46]. For example, pellet formation increases lactic acid and fumaric acid production for *Rhizopus oryzae* [47], while filamentous growth enhances higher α-amylase productivity for *Aspergillus oryzae* [48]. Hence, processes should be designed based on the nature of filamentous fungi and desired products.

In the 5 L shake flask studies, three different inoculum sizes (2%, 5%, and 10%) were studied, considering our preliminary studies. Inoculum size was found to be a non-effective parameter in PBD. Therefore, this parameter was not involved in optimization studies. However, as the scale is enlarged, the inoculum size will also increase, and in parallel, it will cause an increase in cost. Furthermore, inoculum size is directly correlated with pellet formation [43]. Hence, inoculum size was decided to be optimized at this stage. During production in 5 L flasks, filamentous structures formed by fungus were observed. The time-dependent changes in the concentrations of target metabolites measured in 5 L flasks are given in Fig. 4. For the E-AG-01 and E-AG-02, the concentrations of the metabolites increased rapidly from day 2 to 4 and reached equilibrium after day 6 for 2% and 5% of inoculum size (Fig. 4a and b). Higher inoculum size (10%) had a negative effect on E-AG-01 concentration, and the maximum metabolite concentration was determined to be 46.42 mg/L. However, the highest concentrations for E-AG-01 at 2% and 5% inoculum size were 86.21 and 84.43 mg/L on the 10^th^ day, respectively. On the other hand, the metabolite concentration for E-AG-02 was 53.05 and 51.44 mg/L on the 5^th^ day for 2% and 5% inoculum size, respectively, while the metabolite concentration reached 55.18 mg/L at 10% inoculum size on the 8^th^ day. For E-CG-01, the metabolite concentration reached 125.32 and 111.22 mg/L on day 3 at 2% and 5% inoculum size, respectively, while the highest metabolite concentration at 10% inoculum size was 79.25 mg/L on day 4. Consequently, highest concentrations for E-AG-01, E-AG-02 and E-CG-01 were 86.21, 55.18 and 125.32 mg/L, respectively. For E-CG-01 and E-AG-01, 2% inoculum size resulted in the maximum yield, while 10% inoculum size gave the maximum yield for E-AG-02. The biotransformation time for metabolites was consistent with the optimization studies performed in 250 mL shake flasks. The efficiency was increased, 55.3-fold for E-AG-01, 13-fold for E-AG-02, and 1.96-fold for E-CG-01. According to the results, dispersed structures of fungus directly affected the production yield.

*C. laburnicola* catalyzes oxidation, Baeyer Villiger oxidation, ring opening, and dehydration reactions, respectively. There are examples of whole-cell systems that catalyze similar reactions on triterpenoids in the literature. Fungal biotransformation studies on pentacyclic triterpenoids resulted in the production of 3(4)-seco derivatives, whereas lactone formation is less common [49–54]. However, the yields were relatively low in these studies. Shen et al. (2022) reported a biotransformation study on ursane and oleanane-type triterpenoids using *Streptomyces olivaceus* CICC 23628, an actinobacterium. Although 3(4)-seco derivatives were obtained with high yield, hydroxylation reactions occurred afterward in this study [55]. There are biotransformation studies on tetracyclic triterpenoids only with *Glomerella fusarioides*. The study on eburicoic acid is the first example of an A-ring opening reaction [56]. In the biotransformation studies on cycloastragenol and astragenol, lactone and 3(4)-seco derivatives were obtained in low yield, and additional modifications at C-11 were observed [57, 58]. Nevertheless, optimization of whole-cell biotransformation for yield increment was not performed in any of these studies.

## Conclusion

We have discovered that *C*. *laburnicola* can produce biotransformation metabolites from cycloastragenol and astragenol that exhibit impressive telomerase activity (Table S1). This finding has led us to enhance the production of these metabolites. We conducted a study using a design of experiment approach to screen and optimize factors. Through PBD experiments, we found that temperature, biotransformation time, and shaking speed were influential in producing target metabolites. We then performed CCD experiments with these effective parameters and determined the optimal conditions for target metabolites. In 5 L shake flask studies, we obtained significant yield increases for our target metabolites: 55.3-fold for E-AG-01, 13-fold for E-AG-02, and 1.96-fold for E-CG-01. In conclusion, this study demonstrates that the production yield of potent telomerase activators can be increased by optimizing biotransformation parameters, and *C*. *laburnicola*, a newly discovered endophyte with great potential as a biocatalyst, is a good candidate for further industrial utilization.

### Experimental

#### Microorganism and starting compounds

*C. laburnicola* used in this study was isolated from fresh and healthy leaves of *Astragalus angustifolius*. The original strain was deposited at the Bedir Laboratory with the deposit number 20131E4BL1 [37]. All cultures were maintained on potato dextrose agar (PDA, Merck, 1.10130.0500) slants and stored at 4 °C until use. Before biotransformation, the fungus was pre-cultivated on PDA in Petri dishes for ten days at 25 °C. Cycloastragenol (CG) and astragenol (AG) were donated by Bionorm Natural Products (İzmir, Türkiye). The reference compounds, namely E-CG-01, E-AG-01, and E-AG-02, were isolated in our previous study using *C. laburnicola* [37, 39]. The chemical structures of the substrates and target metabolites are presented in Fig. 1.

### Determination of growth curve for *Camarosporium laburnicola*

Stock cultures stored at 4 °C in an agar slant were transferred to the fresh PDA medium and incubated at 25 °C for ten days. Following incubation, Tween 80 solution (0.1%) was added to Petri dishes, and the spore solution was obtained by scraping it with an inoculation loop. After that, the spore solution was thoroughly mixed with a vortex. Erlenmeyer flasks (250 mL) containing 50 mL of potato dextrose broth (PDB, HKM Culture Media, 021053) were prepared for dry weight determination and inoculated with 2% (v/v) spore solution. Each day, nutrient media of three Erlenmeyer flasks were filtered through the Buchner funnel. The remaining cells on the filter paper were dried at 50 °C until a stable weight was measured. The growth curve graph was obtained by changing the dry weight over time.

### Screening and optimization of process conditions

*C. laburnicola* was prepared for biotransformation studies according to the experimental set presented by Design Expert 12. Briefly, Tween 80 solution was added to Petri dishes, and spore solution was obtained by scraping it with an inoculation loop. Then, the spore solution was inoculated into the pH-adjusted PDB medium. Starting compounds (substrates), CG and AG were prepared in DMSO at 10, 20, and 30 mg/mL concentrations. After inoculation, the substrate was dosed at 1% (v/v) of the medium in the substrate feeding time, and production continued under submerged culture in Erlenmeyer flasks (250 mL).

### Screening of process parameters by Plackett-Burman Design

PBD was used to screen and select variables significantly influencing the microbial biotransformation of cycloastragenol and astragenol by *C. laburnicola*. A total of 12 runs of PBD were used to evaluate the nine factors, including substrate feeding time (days), biotransformation time (days), temperature (°C), pH, working volume (mL), shaking speed (rpm), inoculum size (v/v %), substrate concentration (w/v %), and Tween 80 concentration (v/v %). The values used in our previous biotransformation studies were defined as the central point (0) [37, 38, 59]. The parameters’ upper (+ 1) and lower limits (-1) were determined by considering the optimization studies for fungal biotransformation in the literature and the environmental conditions favorable to fungal growth [26, 27, 60, 61]. These factors were tested at the two-level PBD (Table 10). The experimental errors in data analysis were estimated by introducing two unassigned variables, including dummy 1 and dummy 2. Responses of biotransformation product yield (%) were determined by calculating the average value of three replicates measured independently. The statistically significant variables were thus used for further bioprocess optimization.

**Table 10:** Levels of each factor tested in the PBD

Variables	Low (-1)	High (+ 1)
Substrate feeding time (days)	3	5
Biotransformation time (days)	3	9
Temperature (°C)	20	30
pH	4.6	6.6
Working volume (mL)	30	70
Shaking speed (rpm)	150	210
Inoculum size (v/v %)	2	20
Substrate concentration (w/v %)	0.01	0.03
Tween 80 concentration (v/v %)	0	0.2

### Optimization of process parameters by Central Composite Design

CCD was used for optimization studies to obtain the significant effects on the production of telomerase activators and the mutual factor interactions between the selected factors. The optimal value of each variable that significantly influenced the biotransformation process was further identified to increase production yields. Three factors selected from PBD for further optimization were biotransformation time (days), temperature (°C), and shaking speed (rpm). Five different levels of design were employed to evaluate each factor, including factorial points (− 1, + 1), axial points (−α, +α), and central points (0). A total of 18 runs of CCD were performed for the three factors. Table 11 shows the levels of each factor used in the CCD. Constant parameters and their values are given in Table 12.

**Table 11:** Levels of each factor tested in the CCD

Variables	Levels				
	-α	-1	0	+ 1	+α
Biotransformation time (days)	1	3	6	9	11
Temperature (°C)	17	20	25	30	33
Shaking speed (rpm)	130	150	180	210	230

**Table 12:** Parameters and their values are kept constant in the CCD

Variables	Value
Substrate feeding time (day)	3
pH	5.6
Working volume (mL)	50
Inoculum size (v/v %)	10
Substrate concentration (w/v %)	0.02
Tween 80 concentration (v/v %)	0.1

### Scale-up studies

Scale-up studies were carried out using optimized parameter values except for inoculum size. Spore solution was prepared and inoculated at three different levels (2, 5, and 10%) to 1000 mL of PDB medium in 5000 mL Erlenmeyer flasks to produce telomerase activators in shake flasks. Ten mL samples were taken from the shake flasks to measure metabolite concentration.

### Sample preparation and HPLC analysis

The fungal mycelium was filtered from the nutrient medium using a Buchner funnel to terminate the process. Afterward, the broth was extracted three times with ethyl acetate (EtOAc). The organic phase was evaporated using a rotary evaporator. Then, the residue was dissolved in HPLC-grade methanol and filtered using a 0.45 μm PTFE filter.

The content of biotransformation products in the extracts was determined according to standard curves. In brief, reference compounds were dissolved in methanol with a final concentration of 2000 ppm and used as stock solutions. These stock solutions were then diluted by HPLC-grade methanol with seven working concentrations (1250–25 ppm).

HPLC analyses were performed on Agilent Technologies 1200 Series instrument with a quadruple pump, automatic sample injection section, column furnace, and diode array detector (DAD) equipment. A guard column (Security Guard^™^ ULTRA Cartridge UHPLC C18 4.6 mm) and Phenomenex-Kinetex RP column (100 × 2.6 mm; 2.6 μm particle size) were used for separations. HPLC analyses were carried out using the following solvents: 0.1% acetic acid containing ultrapure water (A), 0.1% acetic acid containing acetonitrile (B), and methanol. The gradient elution was performed as 70 A/30B for 2 min, in 6 min to 20 A/80B, kept at that ratio for 2 min, at 10.01 min 100% methanol for 3 min to remove contaminants. Before the next injection, the column was equilibrated for 6 min with the beginning conditions. Injection volume, flow rate, and column temperature for HPLC were 10 µL, 1 mL/min, and 25 °C, respectively. Detection was performed at 200 nm for E-CG-01 and 215 nm for E-AG-01 and E-AG-02. The software used for HPLC analysis was Agilent ChemStation (Rev. B.04.03-SP1).

The yield of target metabolites was calculated with the following equation:

4 $$\begin{array}{l}Target\,metabolite\,yield\,\left( \% \right) = \\{\rm{ }}target\,metabolite\,production\,\left( {mg/L} \right)/substrate\,concentration\\\left( {mg/L} \right) \times 100\% \end{array}$$

### Validation studies

According to the optimum values obtained by PBD − CCD, validation experiments were performed to verify the reliability of the experimental model. The microbial biotransformation studies by *C. laburnicola* were carried out in three replicates, and the resulting values were averaged to obtain final metabolite yields.

### Statistical analysis

All experiments were performed in three replicates, and the data consisted of independent measurements. Design-Expert 12 (trial version, Minneapolis, MN) and GraphPad Prism softwares were utilized for statistical analysis and graph plotting. *p* < 0.05 was considered to be significant.

### Electronic supplementary material

Below is the link to the electronic supplementary material.

Supplementary Material 1

## Data Availability

No datasets were generated or analysed during the current study.
