# Supplementary material for: Optimization of biotransformation processes of Camarosporium laburnicola to improve production yields of potent telomerase activators
Source: Microb Cell Fact. 2024 Jul 10;23:196. doi: 10.1186/s12934-024-02468-0 (PMC11234680; doi:10.1186/s12934-024-02468-0)
Supplement: Supplementary file 1 — Supplementary Material 1 [file 12934_2024_2468_MOESM1_ESM.docx]

**SUPPLEMENTARY INFORMATION**

**Optimization of Biotransformation Processes of *Camarosporium laburnicola* to Improve Production Yields of Potent Telomerase Activators**

Melis Küçüksolak^1^, Hasan Buğra Çoban^2^, Erdal Bedir^1*^

^1^Department of Bioengineering, Faculty of Engineering, İzmir Institute of Technology, 35433, Urla, İzmir, Türkiye

^2^İzmir International Biomedicine and Genome Institute, Dokuz Eylül University, 35340, Balçova, İzmir, Türkiye

***Corresponding Author:**

**Erdal Bedir:** İzmir Institute of Technology, 35430 Urla/İzmir, Türkiye; phone: +90 (232) 750 6952; email: [erdalbedir@iyte.edu.tr](mailto:erdalbedir@iyte.edu.tr)

Table of Contents

[Bioactivity Studies 3](#_Toc170055557)

[Cell Line and Culture Conditions 3](#_Toc170055558)

[Telomerase Activity Assay 3](#_Toc170055559)

List of Tables

[Table S1. Telomerase activity of E-AG-01, E-AG-02, E-CG-01, and CG in cell lysates measured by TeloTAGGG assay 3](#_Toc170055986)

List of Figures

[Fig. S1. Preliminary experiment results for the substrate feeding time. 4](#_Toc170055995)

# **Bioactivity Studies**

## **Cell Line and Culture Conditions**

Primary human epidermal keratinocyte cells (HEKn) (ATCC; PCS-200-010) were cultured in Dermal Cell Basal Media (ATCC; PCS-200-030) supplemented with Keratinocyte Growth Kit (ATCC; PCS-200-040) according to the manufacturer’s instructions at 37°C under humidified 5% CO2. HEKn cells were seeded at a density of 2500 to 5000 cells per cm^2^ when they reached 70-80% confluency.

## **Telomerase Activity Assay**

The identification of telomerase enzyme activity was performed in HEKn cells by TeloTAGGG Telomerase PCR Plus Kit (Sigma Aldrich, 12013789001) according to the supplier’s instruction. The obtained data were presented as a fold change of DMSO used as a solvent control. Experiments were done in two biological experiments with two technical replicates.

**Table S1.** Telomerase activity of E-AG-01, E-AG-02, E-CG-01, and CG in cell lysates measured by TeloTAGGG assay. Values are expressed as the fold change relative to the control DMSO.

|  | **Fold increase versus the control group.** | | | |
| --- | --- | --- | --- | --- |
| **Concentration (nM)** | **E-AG-01** | **E-AG-02** | **E-CG-01** | **CG** |
| 0.5 | 2.65 ± 0.0124 | 9.01 ± 0.0197 | 8.43 ± 0.0200 | 5.07 ± 0.0816 |
| 2 | 1.24 ± 0.0104 | 5.23 ± 0.0063 | 9.35 ± 0.0062 | 5.17 ± 0.1119 |
| 10 | 11.32 ± 0.0145 | 6.04 ± 0,0014 | 9.95 ± 0.0078 | 4.89 ± 0.0617 |
| 30 | 1.93 ± 0.0128 | 7.86 ± 0.0064 | 4.60 ± 0.040 | 3.24 ± 0.0489 |


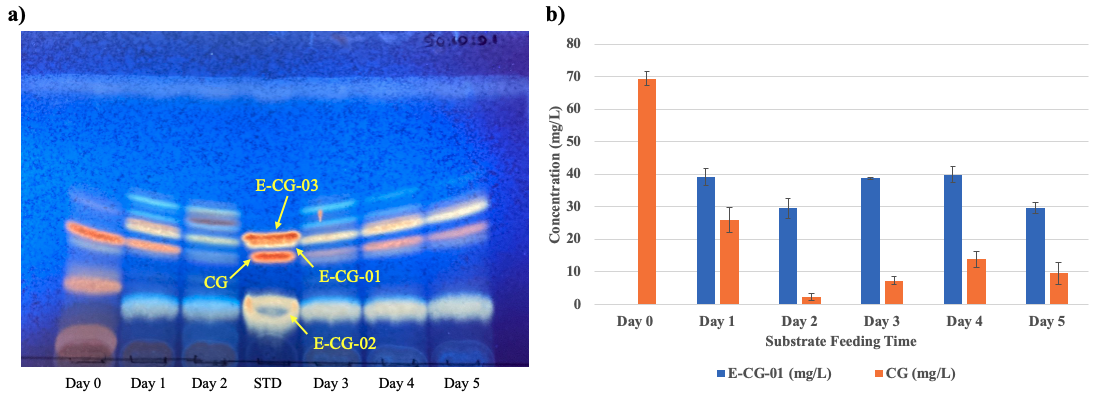


**Fig. S1.** Preliminary experiment results for the substrate feeding time. a) Thin layer chromatogram showing that fungal secondary metabolites and biotransformation products diversify at different substrate feeding times. Normal phase silica gel was used for thin layer chromatography [Mobile phase: 90:10:0.1 (Chloroform:Methanol:Water)]. E-CG-02 and E-CG-03 were other biotransformation products of Camarosporium laburnicola. b) Change of E-CG-01 (one of the target metabolites) and CG (one of the starting compounds) concentrations at different substrate feeding times.
